# Computational Screening of Phase-separating Proteins

**DOI:** 10.1016/j.gpb.2020.11.003

**Published:** 2021-02-19

**Authors:** Boyan Shen, Zhaoming Chen, Chunyu Yu, Taoyu Chen, Minglei Shi, Tingting Li

**Affiliations:** 1 Department of Biomedical Informatics, School of Basic Medical Sciences, Peking University Health Science Center, Beijing 100191, China; 2 Institute of Systems Biomedicine, School of Basic Medical Sciences, Peking University Health Science Center, Beijing 100191, China; 3 MOE Key Laboratory of Bioinformatics, Bioinformatics Division and Center for Synthetic & Systems Biology, BNRist, School of Medicine, Tsinghua University, Beijing 100084, China

**Keywords:** Phase separation, Prediction, Protein–protein interaction, Protein post-translational modification, Immunofluorescence image

## Abstract

**Phase separation** is an important mechanism that mediates the compartmentalization of proteins in cells. Proteins that can undergo phase separation in cells share certain typical sequence features, like intrinsically disordered regions (IDRs) and multiple modular domains. Sequence-based analysis tools are commonly used in the screening of these proteins. However, current phase separation predictors are mostly designed for IDR-containing proteins, thus inevitably overlook the phase-separating proteins with relatively low IDR content. Features other than amino acid sequence could provide crucial information for identifying possible phase-separating proteins: **protein–protein interaction (PPI)** networks show multivalent interactions that underlie phase separation process; **post-translational modifications (PTMs)** are crucial in the regulation of phase separation behavior; spherical structures revealed in **immunofluorescence (IF)images** indicate condensed droplets formed by phase-separating proteins, distinguishing these proteins from non-phase-separating proteins. Here, we summarize the sequence-based tools for predicting phase-separating proteins and highlight the importance of incorporating PPIs, PTMs, and IF images into phase separation **prediction** in future studies.

## Introduction

Cellular organelles can be categorized into two classes, membrane-bound organelles and membraneless organelles. Membrane-bound organelles include classic organelles such as the Golgi apparatus, mitochondrion, and lysosome. These cellular compartments enclosed by lipid bilayers have been well studied in the last century. However, in living cells, many biochemical reactions take place in membraneless organelles [1], [2]. The formation mechanisms and functions of membraneless organelles had remained perplexing until ten years ago. In 2009, Brangwynne et al. reported liquid-like behaviors of P granules, which are protein-rich membraneless organelles in the cytoplasm of cells from *Caenorhabditis elegans* [3]. These granules can flow, deform, and undergo fission freely, just like liquid droplets. Proteins within P granules are also highly mobile and can exchange rapidly with the surrounding cytoplasm. These findings suggest that liquid–liquid phase separation (LLPS, also called liquid–liquid demixing) could be one of the mechanisms underlying membraneless organelle formation.

In a phase separation process, a set of macromolecules such as proteins and nucleic acids are separated from their surrounding environments and form an independent phase. The separated phase shares a similar molecular composition with the surrounding environment, yet at different concentrations [4]. In cells, proteins or nucleic acids form separated phases via intra- or intermolecular interactions [1], [4], [5], thereby allowing the formation of phase-separated compartments, which are also named membraneless organelles or biomolecular condensates. Besides P granules [3], the nucleoli [1], [2], centrosomes [6], stress granules [7], [8], and processing bodies (P-bodies) [8], [9] are also membraneless organelles formed through phase separation. In addition, phase separation underlies many biological processes such as translation regulation [10], [11], mRNA deadenylation [10], [12], heterochromatin formation [13], [14], and the control of signal transduction [15], [16], [17].

Phase separation is a complex biophysical process. Changes to any property of the system, *e.g.*, molecular composition, temperature, electrostatic property, and viscoelasticity of the solution, may affect the phase separation process [1], [5], [18]. Being able to undergo phase separation under specific conditions may be a universal property of proteins. However, only a few proteins with specific sequence-dependent features have the potential to undergo phase separation in living cells [19]. In this review, we name these proteins phase-separating proteins. Scientists have found that certain sequence features may correlate with phase separation behaviors, which brings out a range of useful bioinformatics tools to predict phase-separating proteins. Herein, we summarize the sequence-based predicting tools for phase-separating proteins and integrate the available phase-separating protein data to evaluate their performances. Furthermore, we propose protein–protein interaction (PPI) networks, post-translational modifications (PTMs), and immunofluorescence (IF) images as three promising features to be incorporated into phase separation prediction in future studies.

## Driving force of phase separation

Phase separation is a conditional process. Proteins indispensable to the formation of one condensate can be alternative in another or do not participate in phase separation under some conditions [19]. Multivalent interactions between condensate components are the driving force of phase separation [17], [20], [21].

**Figure 1:**
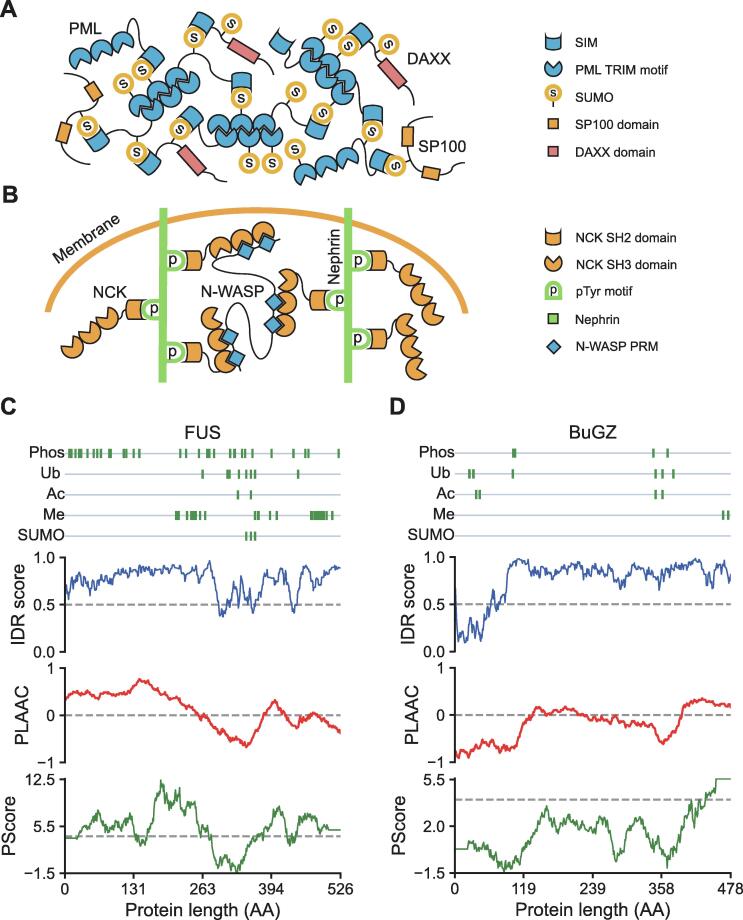
**Schematic view of multivalent interactions that promotephase separation** **A.** PML protein can not only self-assemble via interactions between its TRIMs but also interact via its SIM with SUMOs of itself and other proteins, such as DAXX and SP100. **B.** Schematic view of nephrin–NCK–N-WASP system. The cytoplasmic tail of nephrin contains three pTyr sites, each of which can bind to an SH2 domain on NCK. The three SH3 domains on NCK can also bind to PRMs within N-WASP. **C.** Residue-wise plot of scaffold protein FUS. Information of PTM sites was collected from the PhosphoSitePlus database; IDR scores were predicted by IUPred for long disorder; the prion-like domain was identified with PLAAC score greater than zero; the pi-contact was identified with PScore greater than four, as indicated by dashed line. **D.** Residue-wise plot of scaffold protein BuGZ. PML, promyelocytic leukemia; TRIM, tripartite motif; SIM, SUMO-interacting motif; SUMO, small ubiquitin-like modifier; DAXX, death domain-associated protein; SP100, nuclear autoantigen Sp-100; NCK, non-catalytic region of tyrosine kinase; N-WASP, neuronal Wiskott–Aldrich syndrome protein; pTyr, tyrosine phosphorylation; SH, Src homology; PRM, proline-rich motif; FUS, fused in sarcoma; PTM, post-translational modification; IDR, intrinsically disordered region; BuGZ, BUB3-interacting and GLEBS motif-containing protein ZNF207; Phos, phosphorylation; Ub, ubiquitination; Ac, acetylation; Me, methylation; AA, amino acid.

Proteins able to form multivalent interactions that promote phase separation can be classified into two types: one characterized by multiple modular domains and the other characterized by intrinsically disordered regions (IDRs). The first type of proteins often carry several folded interaction domains. The driving force of phase separation of these proteins is the multivalent interactions between their interaction domains [4]. An example is the interaction between small ubiquitin-like modifier (SUMO) and SUMO-interacting motif (SIM), which plays an essential role in the overall architecture of promyelocytic leukemia (PML) body. The PML protein contains a SUMO-interacting motif and multiple SUMOylation sites [22]. As the scaffold protein of the PML body, PML can not only self-assemble via interactions between its tripartite motifs (TRIMs) but also interact with itself and other proteins via SUMO–SIM interactions [23], [24] (Figure 1A). Another example is the multivalent interactions of the nephrin–non-catalytic region of tyrosine kinase (NCK)–neuronal Wiskott–Aldrich syndrome protein (N-WASP) system in the actin regulatory signaling pathway. Nephrin is a transmembrane protein, the cytoplasmic tail of which harbors three tyrosine phosphorylation (pTyr) sites. Each of these three pTyr sites can bind to a Src homology 2 (SH2) domain on NCK. N-WASP contains six proline-rich motifs (PRMs), which can also bind to three of the SH3 domains on NCK. These interactions further stimulate actin assembly and phase separation [17] (Figure 1B).

The other type of phase-separating proteins are characterized by the presence of IDRs [25], [26]. Instead of having a fixed three-dimensional structure, IDRs can interconvert between a range of slightly different low-energy states with discrepant conformations [27], [28], [29]. Proteins with high proportions of IDRs are hot targets in phase separation studies, *e.g.*, the neurodegenerative disorder-related protein fused in sarcoma (FUS) [30], [31], [32], [33] (Figure 1C), the BUB3-interacting and GLEBS motif-containing protein BuGZ [34], [35] (Figure 1D), and the microtubule-associated protein Tau [36], [37], [38]. However, not all IDRs facilitate phase separation. Phase-separating IDRs often share some sequence features. One type of IDRs that often undergo phase separation is the IDRs encompassing low-complexity regions (LCRs), which are regions with biased amino acid composition, often containing repeated segments. Moreover, phase-separating IDRs are often enriched with specific amino acid residues, *e.g.*, arginine and glycine for the RGG/RG domains [39], [40] or polar amino acid residues like serine, tyrosine, glutamine, and asparagine for the prion-like domains [41]. These amino acids do not appear randomly, but are often found as functional sites like short/eukaryotic linear motifs and alternating charge blocks [25], [42].

## IDR content analysis in the prediction of phase-separating proteins

IDR-containing proteins account for a large proportion of phase-separating proteins, although our understanding of how IDRs are involved in the phase separation process and what features these IDRs share is still limited [43]. Therefore, IDR content analysis is often utilized in bioinformatics screening of potential phase-separating proteins (Table 1, [44], [45], [46], [47], [48], [49], [50], [51]). DisProt is one of the earliest and most influential disorder databases [44], [45]. Two long-tested IDR predictors, ESpritz [46] and IUPred [47], [48], are frequently used in the IDR content analysis of potential phase-separating proteins. In recent years, meta-IDR predictors like D2P2 [49] and MobiDB [50], [51], which integrate predictions from several predictors currently available and provide comprehensive results, are widely used as well. For example, in a previous study on how intrinsically disordered linkers influence the interplay between phase separation and gelation, researchers used the meta-IDR predictor D2P2 to identify proteins containing disordered regions from the human proteome [52].

**Table 1:** **IDR content analysistools**

*Note*: IDR, intrinsically disordered region.

## Phase separation predictors

Although multivalent, transient interactions that drive phase separation are often facilitated by IDRs [21], only proteins with certain types of IDRs facilitate phase separation. Non-IDR interacting elements like the coiled-coil domain can also promote these interactions [6]. Bioinformaticians thus aim to develop prediction tools that are based explicitly on phase separation-specific sequence features.

By studying the sequences of several well-known phase-separating proteins, *e.g.*, FUS, DEAD box protein 4 (DDX4), TATA-binding protein-associated factor 15 (TAF15), Ewing sarcoma protein (EWS), TAR DNA-binding protein 43 (TDP43), and heterogeneous nuclear ribonucleoprotein A1 (HNRNPA1), two common types of LCRs, prion-like domains (PLDs) and RGG domains, were found to promote weak interactions and thereby promote phase separation [33], [39], [53], [54]. Furthermore, low-complexity aromatic-rich kinked segments (LARKSs) and steric zipper motifs have been found to promote the transition between different physical properties of biomolecular condensates [55], [56]. Such features have inspired bioinformaticians to design algorithms to predict the phase separation propensities of proteins [57]. These include PScore [58], prion-like amino acid composition (PLAAC) [59], PSPer [60], catGRANULE [61], R+Y [41], LARKS [55], and ZipperDB [56] (Table 2, [41], [55], [56], [58], [59], [60], [61]).

**Table 2:** **Phase separationanalysis tools**

*Note*: PLD, prion-like domain; FUS, fused in sarcoma; R, arginine; G, glycine; F, phenylalanine; Y, tyrosine; LARKS, low-complexity aromatic-rich kinked segment.

**PScore**

Pi–pi interactions can occur between protein sequences enriched in pi-orbital-containing residues. In contrast to the conventional view, pi–pi interaction involves not only amino acids with an aromatic ring (tyrosine, phenylalanine, tryptophan, and histidine) [62], but also non-aromatic amino acids with pi bonds on their side chains (glutamine, asparagine, glutamic acid, aspartic acid, and arginine) and small amino acids with exposed backbone peptide bonds (glycine, serine, threonine, and proline). Face-to-face interactions formed by these pi-containing groups are called planar pi–pi contacts. In 2018, the Forman-Kay group reported that planar pi–pi contact represents a predominant interaction type and is highly relevant to self-association and phase separation of proteins [58]. Then a planar pi–pi contact predictor named PScore was developed for screening potential phase-separating proteins.

**PLAAC**

PLAAC is an application initially designed to screen PLDs [59], which utilizes a hidden Markov model (HMM) for the discrimination of PLDs and non-PLDs based on amino acid composition. PLAAC was originally trained on yeast proteome but later extended to screen human proteins [63], [64]. It supports both single-protein and proteome scanning.

**PSPer**

PSPer is a rule-based model developed for screening prion-like RNA-binding phase-separating proteins. Expected properties of the FUS-like phase-separating regions are used to build an HMM-like model [41], which identifies PLDs, RNA-recognition motifs, and disordered, arginine-rich regions within a protein [60].

**catGRANULE**

catGRANULE is a phase separation prediction algorithm with good performance for predicting dosage-sensitive proteins [61]. This tool is based on the discovery that cellular toxicity mechanisms of some dosage-sensitive proteins in yeast are well-explained by LLPS theory, as these proteins take part in the formation of cytoplasmic foci in a concentration-related manner. Further studies reveal that these proteins have an increased nucleic acid binding propensity. catGRANULE was therefore developed to screen these proteins by combining nucleic acid binding propensities, structural disorder, sequence length, and content of arginine, glycine, and phenylalanine. Although initially trained against the yeast proteome, catGRANULE has been successfully applied to mammalian and even human proteomes [37].

**R + Y**

R + Y is a predictor built upon the analysis of the molecular grammar of FET family proteins, including FUS, EWS, and TAF15 [65]. In 2018, the Hyman, Alberti, and Pappu groups reported that phase separation behaviors of FET family proteins are determined by interactions between the tyrosine-rich PLDs and the arginine-rich RNA-binding domains [41]. Further analyses indicate that the numbers of tyrosine and arginine residues are inversely correlated with the measured saturation concentrations for phase separation. Extrapolating these findings to the prediction of non-FET proteins, researchers developed the R + Y predictor and utilized it for a human proteome-wide analysis, which has remarkable prediction performance on DNA- and RNA-binding proteins (RBPs).

**ZipperDB**

ZipperDB is a database that includes predicted fibril-forming segments from more than 20,000 putative amyloid-forming protein sequences [56]. Fibrils are highly ordered aggregates characterized by a “steric zipper” structure, whose formation is an essential step in amyloidosis. Amyloid deposition is occasionally observed in cells. Recent evidence indicates that the formation of such deposits could be attributed to a liquid-to-solid phase transition or an atypical phase separation process that forms solid-state compartments. ZipperDB utilizes an algorithm named 3D profiling to analyze the probability of forming a steric zipper structure for every hexapeptide segment.

**LARKS**

LARKSs share similar structural characteristics with steric zippers yet have lower binding energy, as aromatic residues predominate the kinks and affect LARKS stability through weak interactions. LARKSs are characterized by the stacking kinked β sheet pairs, which promote the formation of amyloid fibrils and hydrogels during phase transition. Similar to ZipperDB, 3D profiling was utilized to identify potential LARKSs from inquiry protein sequences. After querying the human proteome of 20,120 proteins, a list of 400 proteins with the most enriched LARKS was provided [55].

In summary, all current phase separation prediction tools are developed based on sequence-dependent features. PScore calculates the pi-contact propensities of each residue in a given protein sequence. PLAAC and PSPer identify specific domains like PLDs based on HMM. catGRANULE calculates the granule propensity of each residue in a given sequence. R + Y calculates the number of tyrosine and arginine residues within disordered regions of a given sequence. LARKS and ZipperDB adopt an algorithm named 3D profiling to measure the probability of a given sequence to fold into a LARKS or a steric zipper.

## Performance evaluation of phase separation predictors

Vernon et al. have recently reviewed several phase separation predictors and compared their prediction performance comprehensively [57]. They find that since each algorithm predicts different kinds of interactions and sequence features, very different protein categories are covered by these predictors. The only exceptions are RBPs, as high prediction confidence is obtained from all predictors for RBPs. However, due to the insufficient phase-separating protein data available, evaluations could only be made on a set of 30 human proteins [57].

At least four LLPS protein databases were released until 2020, including LLPSDB [66], PhaSePro [67], PhaSepDB [68], and DrLLPS [69] (Table 3, [66], [67], [68], [69]). LLPSDB collects *in vitro* data on LLPS-related proteins. The current version of LLPSDB includes 295 independent proteins, which are integrated into 1192 entries with corresponding experimental phase separation conditions. PhaSePro provides the experimental data on 121 proteins driving phase separation in living cells, which is less focused on *in vitro* phase separation conditions and contains a broader array of information than LLPSDB. PhaSepDB contains less detailed information than either LLPSDB or PhaSePro, but provides a larger set of data with 2914 non-redundant proteins localized in more than 30 different organelles, which comprise PubMed-reviewed data, UniProt-reviewed data, and high-throughput data. DrLLPS contains 9285 curated proteins that are known to be associated with LLPS, including 150 scaffold proteins, 987 regulator proteins, and 8148 potential client proteins. All four databases extensively reference the original literature, allowing the user to verify information or conduct further scrutinization.

**Table 3:** **Information offour LLPS databases**

*Note*: LLPS, liquid–liquid phase separation

With the availability of high-quality data provided by the four databases mentioned above, a more comprehensive comparison of phase separation predictors is possible. We constructed a non-redundant positive set (set P) of 278 human proteins involving 90, 59, 233, and 86 proteins from LLPSDB, PhaSePro, the PubMed-reviewed part of PhaSepDB, and the scaffold part of DrLLPS, respectively (Table S1). The remaining majority of human proteome was collected from UniProt and defined as the negative set (set N), which includes 20,227 proteins (Table S1).

To compare the prediction performance of available phase separation predictors, we scored the proteins in both set P and set N using PScore, PLAAC, catGRANULE, and PSPer, which provide online/offline batch prediction (Table S2). For R + Y and LARKS without prediction tools, R + Y scores of 2657 human proteins and 400 human proteins enriched with LARKSs were downloaded from the resepctive websites (Table S2). ZipperDB provides neither batch prediction tools nor bulk download. As a result, we collected prediction scores from six phase separation predictors (Table S2).

**Figure 2:**
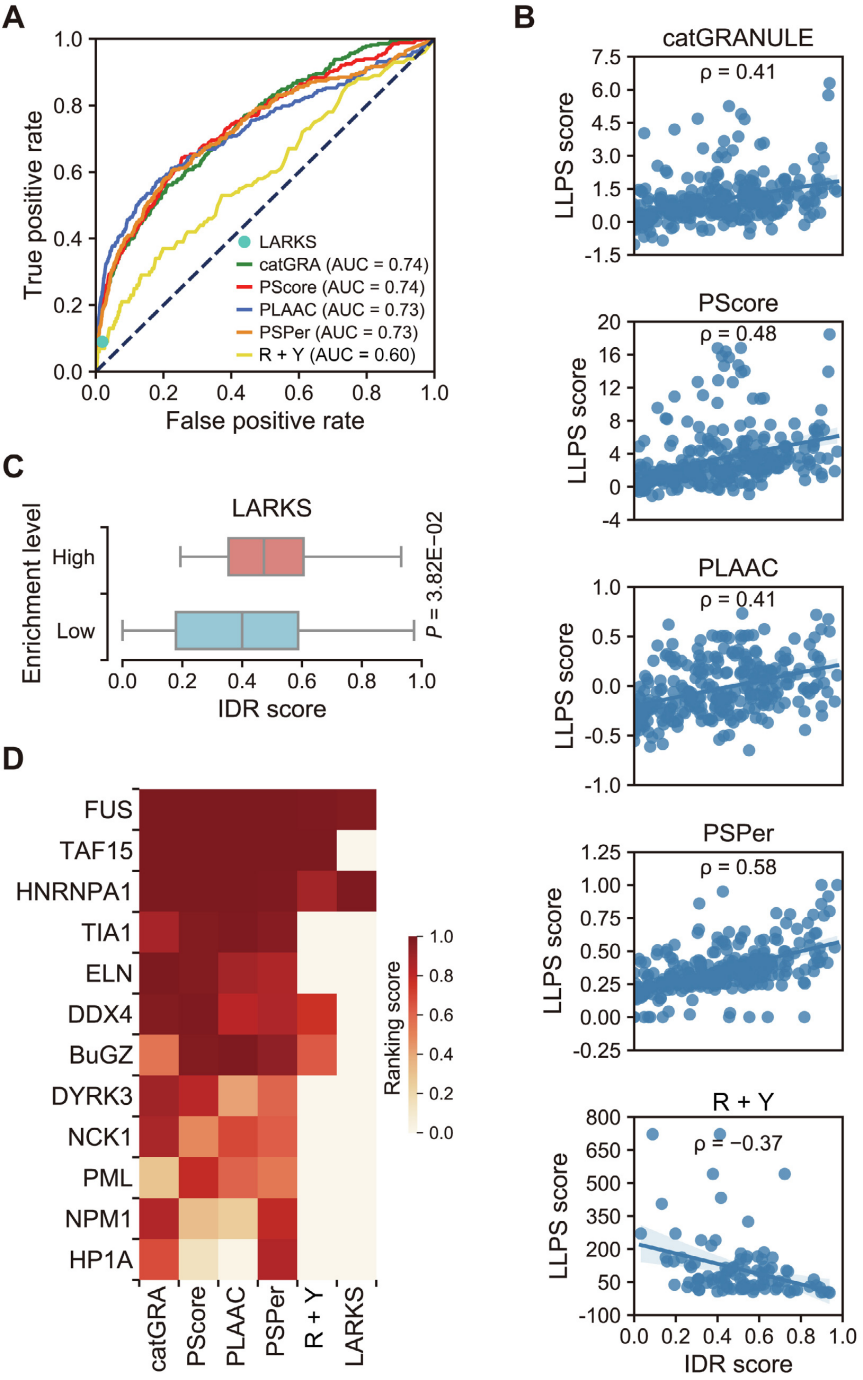
**Comparison of phase separation predictors on human proteome** **A.** ROC curve for each predictor. Since PScore, PLAAC, and PSPer have restrictions on the length of protein sequence, only catGRANULE returned scores for all human proteins. Except for catGRANULE, the AUC scores of the remaining tools were calculated on the subsets of P and N sets. Prediction of LARKS was shown as a point since it did not provide scores for each protein. **B.** Scatter plots of predicted values for proteins in set P, with one axis being the IDR score and the other axis being the phase separation score. Spearman correlation coefficient with *P* < 0.05 indicates significant correlation. **C.** Proteins in set P were divided into LARKS High and LARKS Low groups, according to whether the protein is in the list of LARKS-enriched proteins. *P* value is calculated through the two-sided Mann–Whitney U test. **D.** Ranking scores of 12 specific LLPS proteins by six phase separation predictors. ROC, receiver operating characteristic; AUC, area under the curve; LARKS, low-complexity aromatic-rich kinked segment; LLPS, liquid–liquid phase separation.

Firstly, we adopted a comparison between those six tools on human proteome by plotting the receiver operating characteristic curve (ROC). As shown in Figure 2A, catGRANULE, PScore, PLAAC, and PSPer have nearly the same values for area under the curve (AUC), which are > 0.7, while the performance of R + Y and LARKS is not as good as that of the other four tools.

To test the influence of IDR contents on the scores of these phase separation predictors, we scored the proteins in set P by IUPred with default setting (Table S3). As shown in the scatter plots, phase separation scores of the first five predictors are significantly correlated with IDR scores (Figure 2B). In R + Y, a lower score means a higher phase separation potential, and therefore its prediction scores are significantly negatively-correlated with IDR scores. For LARKS that did not provide prediction scores, proteins in set P were divided into LARKS High and LARKS Low groups, according to whether the protein is in the list of LARKS enriched proteins. The IDR scores of proteins in the LARKS High group were significantly higher than those in the LARKS Low group (Figure 2C). The aforementioned comparison processes were also performed for all human proteins, scores of all phase separation predictors are significantly correlated with IDR scores (Figure S1). These results demonstrate that all phase separation predictors prefer proteins with high IDR contents.

Discriminating the scaffold proteins from the client proteins remains challenging. Therefore, it is possible that some low IDR proteins in set P are client proteins. However, taking the 12 phase-separating proteins for example, heterochromatin protein 1 homolog alpha (HP1A), nucleophosmin 1 (NPM1), PML, and NCK1, which are all scaffold proteins, were ranked outside of the top 10% of the human proteome by all the six predictors, while PML was ranked outside of the top 20% (Figure 2D).

In summary, a range of useful bioinformatics tools have been developed to predict phase-separating proteins, however, most of which are designed for screening IDR-containing proteins. Proteins with modular domains account for a considerable part of phase-separating proteins as well, yet a corresponding computational tool to identify such proteins is not available. It may not be so challenging to identify proteins with multiple modular interaction domains. However, some proteins with few modular domains can also participate in phase separation via assembly, like HP1A [13]. More comprehensive features besides sequence composition might be required for phase separation predictors.

## Other features potentially used to predict phase-separating proteins

Features other than sequence composition could provide crucial information for identifying possible phase-separating proteins. This section will discuss three new features that could be utilized in phase separation prediction: PPI networks, PTMs, and IF images.

### PPI networks

As described in the preceding section, the driving force of phase separation is the multivalent interactions between molecules. In some cases, especially for those with low IDR contents, proteins cannot undergo phase separation alone. Take the nephrin–NCK–N-WASP system in Figure 1B for example. The pTyr–SH2 and SH3–PRM interactions among cooperated proteins are the driving force of the phase separation process, and NCK cannot phase separate under the conditions with nephrin or N-WASP abscent [17]. Another example is the clustering of T cell receptor (TCR) signaling pathway molecules. Upon TCR activation, the tyrosine kinase ZAP70 phosphorylates the transmembrane protein LAT, and phospho-tyrosines on LAT can bind to the SH2 domain on Grb2. Two SH3 domains within Grb2 further interact with Sos1, and the phosphorylated LAT, Grb2, and Sos1 together form the LAT complex, which can coalesce into T cell microclusters that show phase separation behaviors [15]. Similar to the aforementioned example, components in this TCR pathway cannot phase separate without interactions among cooperated proteins.

One issue of the available phase separation predictors is that they are based on sequence-dependent features of individual proteins. These features may not be sufficient, as phase separation driven by complex modular interaction domains or motifs and other multivalent interactions might be missed. A more comprehensive approach that also considers PPI network information could be helpful. It is hard to integrate PPI networks into phase separation predictors directly. One possible approach is network embedding method such as node2vec [70]. Taking the adjacency matrix of the PPI network as input, node2vec encodes each node in the network as a vector, which can be used for various downstream machine learning tasks. The distance between vectors reflects the similarity of interaction networks of corresponding proteins. However, it should be noted that PPI networks based on experimental evidence usually bias to well-studied proteins. BioPlex database that provides an unbiased mapping of the human PPIs by affinity-purification mass spectrometry might be more appropriate to be incorporated into phase separation prediction [71], [72].

### PTMs

The interactions required for phase separation can be weak interactions or strong interactions reversible on a short timescale [21]. The reversibility can often be regulated by PTMs, like the interaction between the SH2 domain and the pTyr residue in nephrin–NCK–N-WASP and TCR pathways [15], [17]. In these examples, PTMs can regulate the reversibility of a binding event by generating/degenerating a modular interaction domain recognition site [21].

PTMs can also regulate phase separation processes by changing protein physical properties, like the charge state, bulkiness, solubility, hydrophobicity, or binding affinity [10], [73], [74], [75]. Citrullination of RG/RGG motifs has been reported to increase the solubility of proteins like FUS, EWS, and TAF15, inhibiting the arginine methylation, aggregation, and stress granule formation of these proteins [76]. Another example is the PTMs of fragile X mental retardation protein (FMRP) and cytoplasmic activation- and proliferation-associated protein 1 (CAPRIN1). The two proteins do not co-phase separate without phosphorylation. However, co-phase separation occurs when Tyr of CAPRIN1 aromatic-rich regions is phosphorylated, or the C-terminal LCR of FMRP is phosphorylated [10], [74].

**Figure 3:**
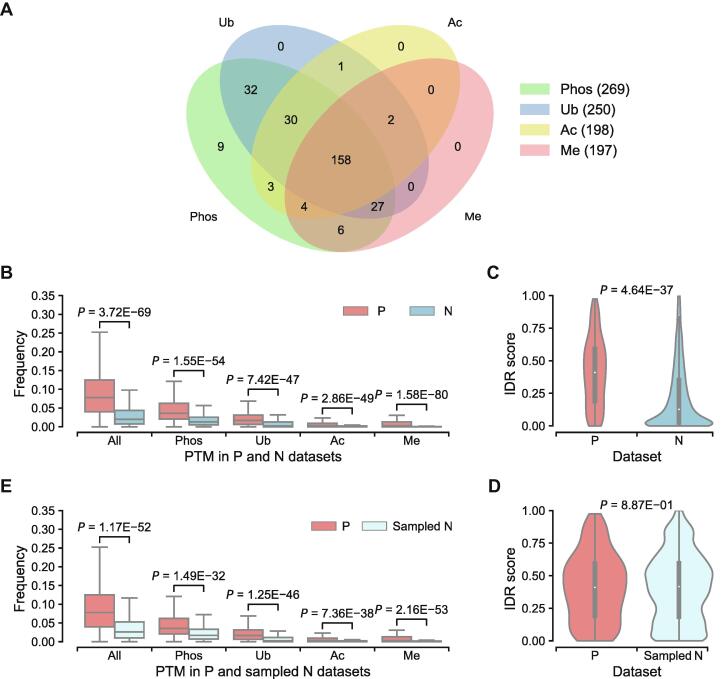
**Comparison of PTM frequencies between different groups on human proteome** **A**. Venn diagram displaying the overlap of phase-separating proteins with different PTM types. The PTM datasets were collected from the PhosphoSitePlus database. **B.** For all PTM types, frequencies for proteins in set P are significantly higher than those for set N. *P* values are calculated through the two-sided Mann–Whitney U test. **C.** Distribution of IDR contents of proteins in set P and set N. Most proteins in set N have low proportions of IDRs. **D.** Set N was resampled into a subset according to IDR content distribution of set P. *P* value indicates that IDR contents of proteins in sampled set N have a similar distribution with that in set P. **E.** PTM frequencies of proteins in set P are still higher than those in resampled set N.

Given the widespread regulatory roles of PTMs in phase separation, we tested whether PTM levels could be used as features to discriminate phase-separating proteins. We collected PTM datasets from the PhosphoSitePlus database [77], including phosphorylation, ubiquitination, acetylation, and methylation (Table S4). As shown in Figure 3A, the Venn diagram displays the overlap of phase-separating proteins with different PTM types. For 278 phase-separating proteins in set P, 221 of them contain more than three types of PTMs, and 158 of them possess all four types of PTMs considered. The overlap demonstrates that most phase-separating proteins are modified by multiple types of PTMs. Then we defined the PTM frequency of a protein as the number of modification sites on a sequence divided by the length of the sequence, and calculated PTM frequencies of proteins in set P and set N. For each protein, frequency of all PTM types and frequencies of each specific PTM types with more than 10,000 recorded sites in the database were calculated (Table S5). For all PTM types, frequencies for proteins in set P were significantly higher than those in set N (Figure 3B). However, it is well established that PTM sites are enriched in IDRs, and proteins with high proportions of IDRs tend to have higher PTM frequencies (Figure 3C), which might cause bias. To control the impact of different IDR contents, the set N was resampled into a subset, whose distribution of IDR contents was similar to that of the set P (Figure 3D; see details of the resampling process in Table S6). The PTM frequencies of the set P were still significantly higher than those of the resampled set N (Figure 3E), which indicates that PTM frequency can be regarded as a feature to discriminate phase-separating proteins. Previous studies reported that an increased number of PTMs was correlated with an increased LLPS propensity in predicted phase-separating proteins [10], [58], and our results show the similar observations using the actual LLPS proteins from more datasets.

It should be noted that although a considerable number of PTM sites have been collected into the database, most of them are phosphorylation sites. Poor data quality and low coverage of the PTM dataset might affect the performance of incorporating PTM frequency as a feature. A high-confidence dataset, including 119,809 phosphorylation sites, has been reported recently [78], which could provide a high-quality PTM feature for identifying possible phase-separating proteins.

### IF images

Identification of spherical droplet structures through IF images represents the most common approach for validation of phase-separating proteins. Phase-separating proteins usually appear as spherical droplet-like structures in IF images, which allows them to be distinguished from non-phase-separating proteins. Thus phase-separating proteins can be identified if we screen out the proteins which appear as spherical structures in IF images. The Cell Atlas (part of the Human Protein Atlas) database provides antibody-based profiling by IF confocal microscopy for 12,073 proteins [79], allowing the screening of phase-separating proteins based on IF images.

It should be noted that the formation of phase separation is condition-dependent. Phase separation proteins that do not appear as droplets in the inquiry IF images cannot be identified. In addition, some phase-separated condensates are too small to be detected in IF images, such as the “transcription hubs” in transcriptional activation [80], and some membrane-bound organelles like vesicles have similar spherical appearance in IF images. Therefore, IF images alone are insufficient to determine whether a specific protein can undergo phase separation or not. However, IF images with droplet-like structures could provide evidence that the labeled proteins aggregate in cells, which allows us to screen out the potential phase-separating proteins. In a previous study, we built a convolutional neural network (CNN) classifier to identify IF images with droplet-like structures and found that the aggregation evidence extracted from IF images is useful in screening phase-separating proteins with low IDR contents [81]. Besides deep learning methods, CellProfiler [82] that can segment droplets and cells in the IF images should also be useful in extracting aggregation evidence from IF images. The outputs of both the CNN classifier and the CellProfiler can measure the aggregation state of the labeled proteins in IF images, and these outputs can be used as features for various downstream machine learning tasks.

## Conclusion and perspectives

Experimental studies have enabled significant progress in improving our understanding of phase separation. Researchers have also noticed that some sequence features are closely related to the phase separation behavior, and several sequence-based computational tools have been developed accordingly. These computational methods facilitate studies on the phase separation phenomenon by providing predictions and proteome-scale screening of phase separation candidates. Furthermore, to examine the roles of different domains of specific proteins in phase separation, truncation mutants are usually constructed to detect segments crucial in forming liquid-like droplets. Computational methods that provide residue-specific predictions also assist phase separation studies in screening critical fragments. However, a meta-predictor that integrates predictions from existing phase separation predictors is in need to reduce the complexity of sequence analysis, like D2P2 and MobiDB in IDR analysis.

Furthermore, current phase separation predictors are mostly designed for IDR-containing proteins and are based on sequence-dependent features of individual proteins. More comprehensive features should be incorporated into phase separation predictors. As multivalent interactions are critical to phase separation, PPI networks can be useful in phase separation computational analysis. PTMs and IF images are also available features that can be utilized in the prediction of phase-separating proteins. PTMs are predominant regulators of phase separation behavior. Accordingly, in this review, we also find that phase-separating proteins tend to have high PTM frequencies. Components of phase-separating condensates usually appear as spherical droplets in IF images, allowing the screening of phase-separating proteins. We expect that incorporating PPI networks, PTMs, and IF images into prediction algorithms will lead to more effective and unbiased phase separation analytic tools.

## CRediT author statement

**Boyan Shen:** Methodology, Investigation, Writing - original draft, Writing - review & editing. **Zhaoming Chen:** Methodology, Formal analysis, Writing - original draft, Writing - review & editing. **Chunyu Yu:** Methodology. **Taoyu Chen:** Writing - review & editing. **Minglei Shi:** Writing - review & editing. **Tingting Li:** Conceptualization, Methodology, Supervision, Writing - review & editing. All authors read and approved the final manuscript.

## Competing interests

The authors have declared no competing interests.

## Supplementary material

Supplementary Figure S1 **Relationships between IDR contents and phase separation predictors on human proteome A.** Scatter plots of predicted values for human proteome, with one axis being the IDR score and the other axis being the phase separation score. Spearman correlation coefficients with *P* values less than 0.05 indicate that the predicted scores of five tools are all significantly correlated with IDR scores. **B.** Human proteins were divided into LARKS High and LARKS Low groups according to whether the protein is in the list of LARKS enriched proteins. The IDR scores of proteins in the LARKS High group were significantly higher than those in the LARKS Low group. *P* value is calculated from the two-sided Mann–Whitney U test.

Supplementary Table S1 **List of proteins in positive set P and negative set N, source databases of proteins in set P are given**

Supplementary Table S2 **Predicted scores of human proteome by phase separation predictors**

Supplementary Table S3 **Predicted IDR scores of human proteome by IUPred**

Supplementary Table S4 **Human modification site datasets downloaded from the PhosphoSitePlus database**

Supplementary Table S5 **PTM frequencies of human proteome**

Supplementary Table S6 **List of proteins in positive set P and sampled negative set N**
