# Supplementary figures and images for: Computational Screening of Phase-separating Proteins
Source: Genomics Proteomics Bioinformatics. 2021 Feb 19;19(1):13–24. doi: 10.1016/j.gpb.2020.11.003 (PMC8498823; doi:10.1016/j.gpb.2020.11.003)

## Slide 1
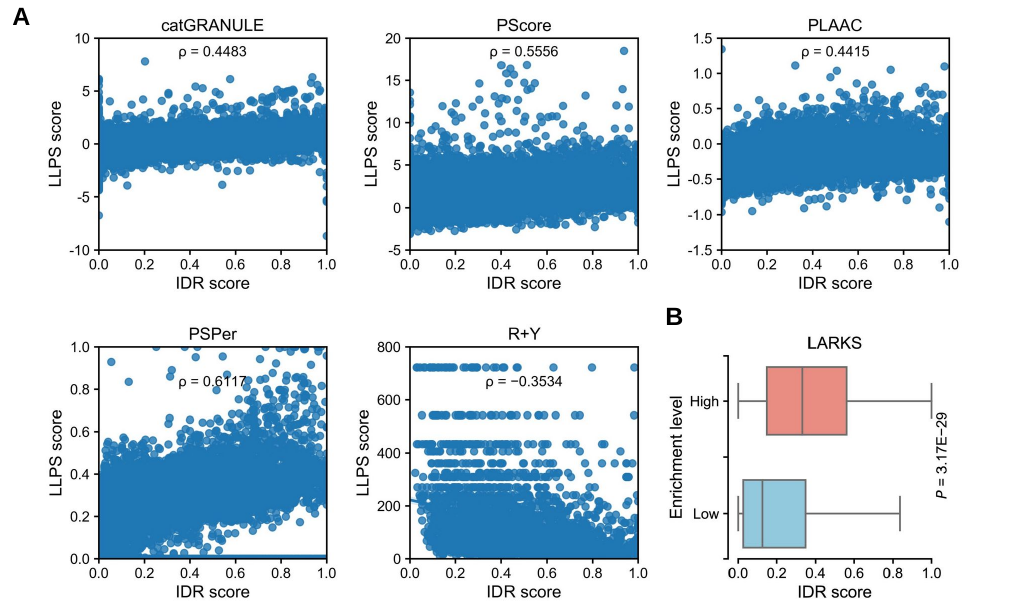

Figure S1
A
B

Supplement: Supplementary Figure S1 — Relationships between IDR contents and phase separation predictors on human proteome A. Scatter plots of predicted values for human proteome, with one axis being the IDR score and the other axis being the phase separation score. Spearman correlation coefficients with P values less than 0.05 indicate that the predicted scores of five tools are all significantly correlated with IDR scores. B. Human proteins were divided into LARKS High and LARKS Low groups according to whether the protein is in the list of LARKS enriched proteins. The IDR scores of proteins in the LARKS High group were significantly higher than those in the LARKS Low group. P value is calculated from the two-sided Mann–Whitney U test. [file mmc1.pptx]
